# The Siderophore Piscibactin Is a Relevant Virulence Factor for *Vibrio anguillarum* Favored at Low Temperatures

**DOI:** 10.3389/fmicb.2018.01766

**Published:** 2018-08-02

**Authors:** Miguel Balado, Marta A. Lages, Juan C. Fuentes-Monteverde, Diana Martínez-Matamoros, Jaime Rodríguez, Carlos Jiménez, Manuel L. Lemos

**Affiliations:** ^1^Department of Microbiology and Parasitology, Institute of Aquaculture, Universidade de Santiago de Compostela, Santiago de Compostela, Spain; ^2^Department of Chemistry, Faculty of Sciences and Center for Advanced Scientific Research (CICA), Universidade da Coruña, A Coruña, Spain

**Keywords:** *Vibrio anguillarum*, siderophores, vanchrobactin, piscibactin, bacterial virulence, fish pathogens

## Abstract

*Vibrio anguillarum* causes vibriosis, a hemorrhagic septicaemia that affects many cultured marine fish species worldwide. Two catechol siderophores, vanchrobactin and anguibactin, were previously identified in this bacterium. While vanchrobactin is a chromosomally encoded system widespread in all pathogenic and environmental strains, anguibactin is a plasmid-encoded system restricted to serotype O1 strains. In this work, we have characterized, from a serotype O2 strain producing vanchrobactin, a novel genomic island containing a cluster of genes that would encode the synthesis of piscibactin, a siderophore firstly described in the fish pathogen *Photobacterium damselae* subsp. *piscicida*. The chemical characterization of this siderophore confirmed that some strains of *V. anguillarum* produce piscibactin. An *in silico* analysis of the available genomes showed that this genomic island is present in many of the highly pathogenic *V. anguillarum* strains lacking the anguibactin system. The construction of single and double biosynthetic mutants for vanchrobactin and piscibactin allowed us to study the contribution of each siderophore to iron uptake, cell fitness, and virulence. Although both siderophores are simultaneously produced, piscibactin constitute a key virulence factor to infect fish, while vanchrobactin seems to have a secondary role in virulence. In addition, a transcriptional analysis of the gene cluster encoding piscibactin in *V. anguillarum* showed that synthesis of this siderophore is favored at low temperatures, being the transcriptional activity of the biosynthetic genes three-times higher at 18°C than at 25°C. We also show that iron levels and temperature contribute to balance the synthesis of both siderophores.

## Introduction

*Vibrio anguillarum* is a bacterium inhabitant of marine environments and also one of the most important pathogens in the aquaculture industry worldwide. It is the causative agent of vibriosis, a fatal hemorrhagic septicaemia affecting warm- and cold-water fish of highly economic importance (Toranzo et al., 2005). *V. anguillarum* isolates are classified into 23 different O-serogroups (O1 to O23) according to the European serotyping system (Pedersen et al., 1999), although most virulent strains belong to serotypes O1, O2 and, to a lesser extent, O3. The remaining *V. anguillarum* serotypes are mostly environmental strains isolated from seawater, marine animals or sediments (Frans et al., 2011; Toranzo et al., 2017).

It is well known that bacterial virulence is a multifactorial trait and that pathogenic bacteria must have a number of factors that enable them to colonize the host and establish an infection (Miller et al., 1989). The virulence-related factors identified in *V. anguillarum* so far include those related to chemotaxis and motility (O’Toole et al., 1996; Ormonde et al., 2000), adhesion (Wang et al., 1998), invasion (Croxatto et al., 2007), secretion of extracellular enzymes (Denkin and Nelson, 2004; Rodkhum et al., 2005; Li et al., 2008) and several iron uptake mechanisms (Li and Ma, 2017). Despite this knowledge, the pathogenesis of *V. anguillarum* is not completely understood (Hickey and Lee, 2017).

Iron restriction is an important host defense strategy, thus successful pathogens must possess mechanisms to acquire iron from host sources in order to cause disease. In *V. anguillarum* two catechol siderophores, vanchrobactin and anguibactin, have been described (Actis et al., 1986; Soengas et al., 2006; Lemos et al., 2010; Li and Ma, 2017). While anguibactin is encoded by pJM1-type plasmids and is restricted to some virulent strains of serotype O1, vanchrobactin is a chromosomally encoded siderophore that is widespread in all *V. anguillarum* isolates either environmental or pathogenic (Lemos et al., 2010; Li and Ma, 2017). It is noticeable that vanchrobactin is not produced by serotype O1 strains harboring a pJM1-type plasmid since the presence of this plasmid results in the inactivation of vanchrobactin synthesis by a transposition event from the plasmid to the vanchrobactin chromosomal gene cluster (Naka et al., 2008). Thus, to date the simultaneous production of more than one siderophore was not reported in *V. anguillarum* (Naka et al., 2008; Rønneseth et al., 2017). However, it was detected the simultaneous presence of anguibactin and vanchrobactin in a marine *Vibrio* sp. (Sandy et al., 2010). From previous reports, it is clear that anguibactin is a key virulence factor for *V. anguillarum* serotype O1 strains (Wolf and Crosa, 1986; Naka et al., 2011). However, the role of vanchrobactin in virulence remains unknown so far.

The *in silico* analysis of the *V. anguillarum* serotype O2 strain RV22 genome sequence (accession number AEZB00000000) showed the existence of a gene cluster whose closest homolog is the *irp* cluster encoding the siderophore piscibactin (Naka et al., 2011). This siderophore was firstly identified in the fish pathogen *Photobacterium damselae* subsp. *piscicida* (Souto et al., 2012) and it is one of the main virulence factors of this bacterium (Osorio et al., 2015). Piscibactin synthesis and transport are encoded by a pathogenicity island (PAI) located in the 69-kb plasmid pPHDP70 (Osorio et al., 2006, 2015). The presence of *irp* genes in *V. anguillarum* RV22 suggests that this strain, besides producing vanchrobactin, likely produces also piscibactin or a piscibactin-like siderophore, which would imply the existence of a third siderophore in *V. anguillarum*.

In this work, we describe a novel genomic island present in many virulent strains of *V. anguillarum* that encodes the piscibactin system. We demonstrate that some serotype O2 strains produce in fact piscibactin and that this siderophore plays a relevant role in the cell fitness and contribute to a greater extent than vanchrobactin to the virulence for fish. We also show that the synthesis of both siderophores is balanced in response to iron levels and to the growth temperature.

## Materials and Methods

### Bacterial Strains, Plasmids, and Media

The bacterial strains and plasmids used are listed in **Table 1**. *V. anguillarum* and *P. damselae* subsp. *piscicida* (*Pdp*) strains were routinely grown at 25°C on tryptic soy agar (TSA) or broth (TSB) (Cultimed) supplemented with 1% NaCl (TSA-1 or TSB-1). *Escherichia coli* strains were routinely grown at 37°C in Luria-Bertani (LB) broth and LB agar (Cultimed). When it was required, antibiotics were added at the following final concentrations: kanamycin (Km) at 50 μg mL^-1^, ampicillin (Ap) sodium salt at 50 μg mL^-1^, and gentamicin (Gm) at 12 μg mL^-1^.

**Table 1:** Strains and plasmids used in this study.

Strain or plasmid	Description	Reference
**Strains**		

***V. anguillarum***		
RV22	Wild-type serotype O2 strain isolated from diseased turbot (Spain)	Lemos et al., 1988
MB14	RV22 with in-frame deletion of *vabF* gene	Balado et al., 2006
MB67	RV22 with in-frame deletion of *vabD* gene	Balado et al., 2008
MB280	RV22 with in-frame deletion of *irp1* gene	This study
MB203	RV22 with in-frame deletion of *vabF* and *irp1* genes	This study
MB299	MB203 carrying pPHDP70::kan	This study
***Photobacterium damselae* subsp. *piscicida (Pdp)***		
DI21	Piscibactin producer strain (DI21 *irp*^+^)	Toranzo et al., 1991
AR84	DI21 cured of plasmid pPHDP70 (DI21 *irp*^-^)	Osorio et al., 2015
***E. coli***		
DH5α	Cloning strain	Laboratory stock
S17-1 λ*pir*	RP4 (Km::Tn7, Tc::Mu-1) *pro-82 λpir recA1 endA1 thiE1 hsdR17 creC510*	Herrero et al., 1990

**Plasmids**		

pWKS30	Low-copy cloning vector, Ap^r^	Wang and Kushner, 1991
pHRP309	Low-copy *lacZ* reporter plasmid, *mob* Gm^r^	Parales and Harwood, 1993
pNidKan	Suicide vector derived from pCVD442; Km^r^	Mourino et al., 2004
pPHDP70::Km	pPHDP70 with a Km^r^ gene inserted contains all necessary genes for piscibactin synthesis	Osorio et al., 2015
pMB12	*vabH* promoter fused to promoterless *lacZ* gene in pHRP309, Gm^r^	Balado et al., 2008
pMB276	*frpA* promoter (P*frpA*) fused to promoterless *lacZ* gene in pHRP309, Gm^r^	This study
pMB277	*araC1* promoter (P*araC1*) fused to promoterless *lacZ* gene in pHRP309, Gm^r^	This study
pML214	*araC2* promoter (P*araC2*) fused to promoterless *lacZ* gene in pHRP309, Gm^r^	This study
pML263	*proC* promoter (P*proC*) fused to promoterless *lacZ* gene in pHRP309, Gm^r^	This study


### Construction of *irp1* Defective Mutants by Allelic Exchange and Complementation

In-frame (non-polar) deletions of *irp1* were constructed by allelic exchange in *V. anguillarum* RV22 wild type and *ΔvabF* (vanchrobactin synthesis deficient) backgrounds using the suicide vector pNidKan as previously described (Balado et al., 2006). The oligonucleotides used are shown in **Table 2**. The mutagenesis process led to the generation of *V. anguillarum* single mutant strains RV22Δ*irp1* (MB280) and the double mutant RV22Δ*vabF*Δ*irp1* (MB283). To ensure that all mutations were in frame, the deleted region was sequenced by the Sanger method using primers *irp1_ang_1_XbaI* and *irp1_ang_4_EcoRI* (**Table 2**). pPHDP70 plasmid (from *Pdp* DI21 and containing all necessary genes for piscibactin synthesis) marked with a Km cassette (pPHDP70::kan) (Osorio et al., 2015) was mobilized into the *V. anguillarum* RV22Δ*vabF*Δ*irp1* double mutant to restore piscibactin synthesis.

**Table 2:** Oligonucleotides used for construction of mutants by allele exchange, transcriptional fusions, and RT-PCR assays.

Oligonucleotide (5′→3′)		Amplification size (bp)
***irp1* mutant construction**		
irp1_ang_1_XbaI	GCTCTAGATGATGCATTAGCCCATCAGG	1489
irp1_ang_2_BamH1	GCGGATCCAAAGCAAGGGTCGAGAGTGT	
irp1_ang_3_BamH1	CGGGATCCAAAGAGCTCAGCCAGATCAC	1360
irp1_ang_4_EcoRI	GCGAATTCTGCAACAGATAATCACCGTG	
***proC* promoter fusion construction**		
1_proC_F_XbaI	GGCTCTAGATGTGCAAGAGGGCGCGTATA	498
2_proC_R_BamH1	CGCGGATCCGGCGACTAAGCCTGCAATAA	
***frpA* promoter fusion construction**		
irp_ang_pr1_F_BamHI	GCGGGATCCACTTTGCCACCCACCATTAC	695
irp_ang_pr1_R_XbaI	GCGTCTAGAATCATGGCCACTTTCGAGTG	
***araC1* promoter fusion construction**		
irp_ang_pr2_F_BamHI	GCGGGATCCGCGATACACTCTTCGTAGTG	664
irp_ang_pr2_R_XbaI	GCGTCTAGAACGTTTCGGTAAGCGTATGG	
***araC2* promoter fusion construction**		
AraC1_F_P_XbaI	CCGTCTAGACTCGCGACTATTTACCAGCA	656
AraC2_P_R_BamHI	GGCGGATCCGATCACACAGCAACGTAACG	

**RT-PCR experiments**		

RT	TTTGGAGATGAGTGCGACAC	
**PCR1**		
araC1_F	GATATGCGCTTTGACTGCCA	196
araC1_R	CTGTGAGACGGCATACAAGC	
**PCR2**		
frpA_F	CGGTGGTAATGCTCAAGGTG	204
frpA_R	TGGCTCGGTAGGTGTTCAAT	
**PCR3**		
irp2_F	AGCAGGCAACAAAGAGTGAG	413
irp1_R	GGGCGAATAACCAAACAAGC	


*Recognition sequences for restriction enzymes are underlined.*

### Growth Promotion and Siderophore Production Assays

Growth promotion assays were performed using 96-well microtiter plates. Each well contained 200 μL of CM9 medium (Lemos et al., 1988) supplemented with 10 μM FeCl_3_ to achieve iron excess conditions, or with the iron chelators EDDA [ethylenediamine-di(*o*-hydroxyphenyl-acetic acid)] 5 μM or 100 μM 2,2′-dipyridyl, to achieve iron restricted conditions. Each well was inoculated with a 1:50 dilution of a *V. anguillarum* overnight culture in TSB-1 adjusted to an OD_600_ = 0.5. The plates were incubated at 25°C or 18°C with shaking at 150 rpm. Growth (OD_600_) was recorded during 24 h in an iMACK Microplate reader (Bio-Rad). Bacterial cultures in CM9 with 50 μM 2,2′-dipyridyl and an OD_600_ ≈ 0.8 (after 6 h of incubation) were used to obtain supernatants and measure siderophore production using the chrome azurol-S (CAS) liquid assay (Schwyn and Neilands, 1987). For this purpose, equal volumes of cell free supernatants and CAS reagent were mixed and, after 15 min of incubation at room temperature, A_630_ was measured in a spectrophotometer (Hitachi).

### Cross-Feeding Assays

To test whether *V. anguillarum* was able to use piscibactin as iron source, a cross-feeding assay was conducted using RV22 (wild type strain) and RV22Δ*vabD* mutant as indicator strains. These strains were inoculated into CM9 plates as follows: 0.5 mL of a TSB-1 culture at an OD_600_ = 0.5 were included into 20 mL of CM9 medium containing 0.8% agarose and 120 μM of the iron chelator 2,2′-dipyridyl, a concentration close to the MIC and at which growth halos are easily visualized (Balado et al., 2006). *V. anguillarum* strains to be tested for piscibactin production and *P. damselae* subsp. *piscicida* DI21 (wild type strain, piscibactin producer) or a cured strain (DI21 lacking pPHDP70, unable to produce piscibactin and used as negative control) were cultured in TSA-1 plates and the cells were harvested with a sterile loop and placed on the surface of the plates inoculated with the *V. anguillarum* strains. The presence of growth halos of the *V. anguillarum* indicator strains around cells of *P. damselae* subsp. *piscicida* DI21 or *V. anguillarum* after overnight incubation at 25°C was indicative of piscibactin utilization.

### Piscibactin Detection by Mass Spectrometry

Siderophore piscibactin detection was carried out in cell free supernatants from cultures of *V. anguillarum* grown at 25°C in flasks containing 1 L of CM9 minimal medium with 40 μM 2,2′-dipyridyl with continuous shaking at 150 rpm for ca. 24 h (Souto et al., 2012). Flasks were inoculated with 20 mL of a fresh culture of *V. anguillarum* RV22wt, RV22Δ*vabF* or RV22Δ*vabD* mutants in TSB-1. When bacterial cultures achieved an OD_600_ = 1.0, 10 mg of GaBr_3_ were added to each 1.0-L batch. After 12 h of incubation at 4°C, bacterial cells were removed by centrifugation at 10,000 ×*g* for 10 min (Beckman J-21 High Speed Centrifuge) and supernatants were filtered through a 0.45-μm pore size membrane by a continuous filtration cartridge (Millipore). Subsequently, ca. 900 mL of each cell-free supernatant were concentrated under vacuum to 410 mL. Fifty milliliters of the solution were passed through an Oasis hydrophilic lipophilic (HLB) cartridge (Waters) (35 cm^3^, 6 g) (Espada et al., 2011), which was previously conditioned and equilibrated with 60 mL of acetonitrile (solvent B) and 60 mL of water (solvent A). They were eluted with 30 mL of the following mixtures of water and acetonitrile: 1:0, 7:3, 1:1, 3:7, and 0:1. The fraction eluted with a mixture of water and acetonitrile (1:1), named RV22WTF3, RV22Δ*vabF*F3, and RV22Δ*vabD*F3, were subjected to high-performance liquid chromatography (HPLC)-high-resolution electrospray ionization mass spectrometry (HRESIMS) analysis using an Atlantis dC18 column (100 mm × 4.6 mm, 5 μm) with a 35 min gradient from 10 to 100% CH_3_CN-H_2_O, then 5 min at 100% CH_3_CN, and finally a 10 min gradient from 100 to 10% H_2_O-CH_3_CN, at a flow rate of 1 mL min^-1^. Piscibactin-Ga(III) complex was detected with a *t_R_* of 9.56 min from RV22WTF3 and with a *t_R_* of 9.71 min from RV22Δ*vabF*F3 by comparison of the *t_R_*, HRESIMS and UV spectral data to those reported for that compound isolated from *P. damselae* subsp. *piscicida* DI21 (Souto et al., 2012). Vanchrobactin was detected with a *t_R_* of 2.74 min from RV22WTF3.

### RNA Purification and RT-PCR

**FIGURE 1:**
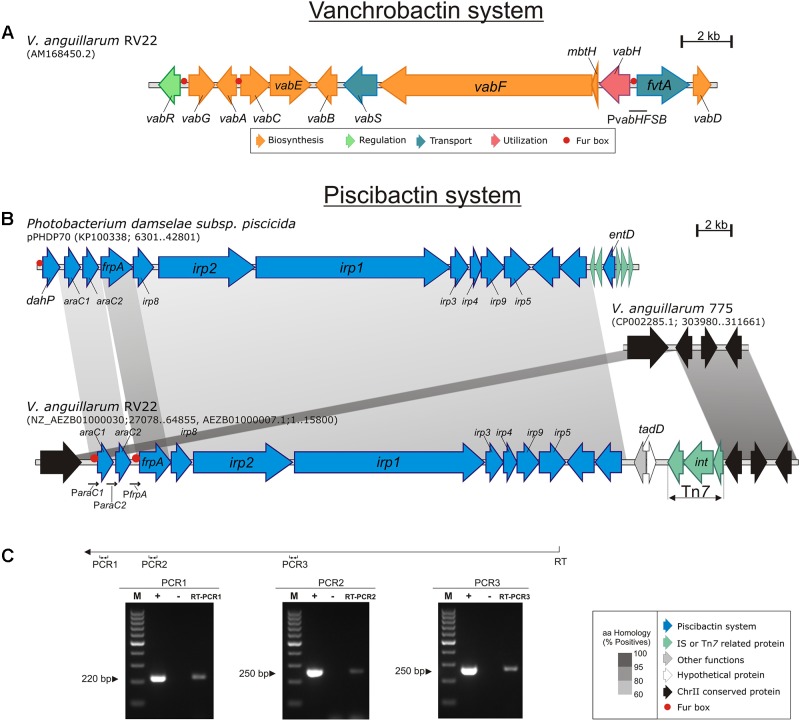
Genetic maps of the gene clusters encoding the vanchrobactin **(A)** and the piscibactin **(B)** systems in *V. anguillarum* RV22. In **(B)** the piscibactin genes, which are part of the plasmid pPHDP70 of *P. damselae* subsp. *piscicida* are included for comparative purposes. Gray blocks indicate percentages of similarity in the proteins sequence. **(C)** Displays the results of three RT-PCRs reactions designed to analyze the transcription of the piscibactin gene cluster in RV22. An appropriate primer (Primer RT, **Table 2**), located at the 3′-end of *irp5* gene, was used to obtain a cDNA (denoted as RT in the figure) that was then used as template for three PCR reactions targeted into *araC1* (PCR1), *frpA* (PCR2) and between *irp2* 3′-end and *irp1* 5′-end (PCR3). M, size marker from 100 to 1,000 bp; +, positive control PCR using genomic DNA as template; -, negative control PCR using total RNA without reverse transcriptase; RT-PCR1-3, results of the three different RT-PCRs.

The organization of piscibactin genes into operon(s) was tested by reverse transcription PCR. *V. anguillarum* RV22 was grown until exponential phase (ca. OD_600_ = 1) in 10 mL CM9 medium containing 5 μM EDDA or 10 μM Fe_2_(SO_4_)_3_. Cells were pelleted by centrifugation at 10,000 ×*g* for 10 min and total RNA was isolated with RNAwiz (Ambion) following the manufacturer’s recommendations. RT-PCR was performed with 1 μg RNA pre-treated with RQ1 RNase-Free DNase (Promega) by using the M-MLV reverse transcriptase (Invitrogen). An appropriate primer (**Table 2**), located at the 3′-end of *irp5* gene, was used to obtain a cDNA that was then used as template for three PCR reactions targeted into *araC1* (PCR1), *frpA* (PCR2) and between *irp2* 3′-end and *irp1* 5′-end (PCR3) (**Figure 1**). All primers used are listed in **Table 2**. A negative control reaction was performed with total RNA without M-MLV reverse transcriptase to confirm the lack of genomic DNA contamination in each reaction mixture. The PCR positive control reaction was done using 100 ng of genomic DNA as template.

### *lacZ* Transcriptional Fusions and β-Galactosidase Assays

DNA fragments corresponding to *V. anguillarum araC1*, *araC2* and *frpA* promoter regions were amplified by PCR. The PCR-amplified promoter regions were fragments of about 700 bp, including the region upstream of the start codon and the first nucleotides of the *araC1*, *araC2* or *frpA* coding sequences (ca. 50 bp). These putative promoter regions were fused to a promoterless *lacZ* gene in the low-copy-number reporter plasmid pHRP309 (Parales and Harwood, 1993). The resulting transcriptional fusion constructs, *araC1::lacZ* (pMB34), *araC2::lacZ* (ML214) and *frpA::lacZ* (pMB33), were mobilized from *E. coli* S17-1 aaa*pir* into *V. anguillarum* strains RV22 wild type or RV22Δ*vabD* mutant by conjugation. The construction *vabH::lacZ* (pMB12), corresponding to the promoter of the vanchrobactin operon (*vabH*) (Balado et al., 2008), was used as reference of a siderophore promoter. A housekeeping gene promoter (P*proC*) was used as control (Savioz et al., 1990). The *V. anguillarum* strains carrying the promoter–*lacZ* fusions or the plasmid pHRP309 alone (negative control) were grown in CM9 minimal medium under different iron-availability conditions. The β-galactosidase (LacZ) activities were measured by the method of Miller (1992). Results showed are means of three independent experiments, each one measured in triplicate.

### *In Silico* Analysis of *irp_ang_* Genomic Island Structure and Analysis of Its Distribution

The NCBI services were used to consult DNA and protein sequence databases using BLAST algorithms. All *V. anguillarum* genome sequences (43 genomes) deposited in GenBank were screened for the presence of the *irp_ang_* genomic island. For this purpose, the whole nucleotide sequence of *irp_ang_* genomic island of *V. anguillarum* RV22 and the 3′ and 5′-ends surrounding regions were used as query in a BLASTN search. Nucleotide sequences producing significant alignments were downloaded and a multiple alignment of the conserved genomic sequences was obtained by using MAUVE (Darling et al., 2010). The alignment was used to compare the genomic island structure and to identify the insertion point. The *Virtual Footprint Promoter Matches* from the online database PRODORIC Release 8.9^1^ (Münch et al., 2003) was used to identify putative Fur-box sequences. BLASTP algorithm was also used to analyze homology of each protein encoded by the *irp_ang_* gene cluster from *V. anguillarum* with its counterpart in *P. damselae* subsp. *piscicida*. All this information was used to create **Figure 1** and Supplementary Figure S1.

### Fish Virulence Assays

Virulence assays were carried out with turbot (*Psetta maxima*) fingerlings with an average weight of 5 g. Fish were divided in groups of 30 animals. All fish groups, one per strain tested and a control group, were maintained in 50-L seawater tanks at 18°C with continuous aeration and water recirculation. The bacterial inoculum was prepared as follows. Several colonies from a 24 h TSA-1 culture were suspended into saline solution (0.85% NaCl) to get a cell suspension with an OD_600_ = 0.5. The inoculum used was a 10-fold dilution of this suspension in saline solution. The actual number of injected bacterial cells was determined by plate count on TSA-1. Fish were inoculated intraperitoneally (ip) with 100 μL of bacterial suspensions, ranging the doses used between 2 and 4 × 10^4^ CFU per fish. A control group was inoculated with 100 μL of saline solution. Mortalities were recorded daily during 10 days after injection and statistical significance of differences in percentage survival for *V. anguillarum* strains was determined using the Kaplan-Meier method with Mantel-Cox log-rank test using SPSS (version 20; IBM SPSS Inc., Chicago, IL, United States). *P*-values were considered significant when *P* was <0.05, <0.01, and <0.001. All protocols for animal experimentation used in this study have been reviewed and approved by the Animal Ethics Committee of the University of Santiago de Compostela.

## Results

### *V. anguillarum* Strain RV22 Harbors a Novel Genomic Island Likely Encoding Piscibactin Synthesis

In a previous work, we described and characterized the gene cluster involved in the synthesis and transport of vanchrobactin system in *V. anguillarum* RV22 (**Figure 1A**) (Balado et al., 2006). Inactivation of vanchrobactin synthesis resulted in a drastic decrease in siderophore production by this strain and in its growth ability under iron starvation conditions. However, when the complete genome sequence of RV22 was described (Naka et al., 2011), it was found that chromosome II contains a likely additional siderophore gene cluster with high similarity to the *irp* genes encoding piscibactin in *P. damselae* subsp. *piscicida* (Souto et al., 2012).

This *irp* gene cluster in *V. anguillarum* RV22 strain (that we named *irp_ang_*) is part of a DNA region of ca. 40 kb located in chromosome II (between locus AEH34691 and AEH34692) (**Figure 1B**). This cluster shows identical gene structure and organization as the *irp* cluster described in *P. damselae* subsp. *piscicida* as part of pPHDP70 plasmid (**Figure 1B**) (Souto et al., 2012; Osorio et al., 2015). Each CDS present in the *irp_ang_* cluster shows an amino acid similarity between 52 and 66% with the *irp* cluster ortholog from *P. damselae* subsp. *piscicida* (Supplementary Table S1). Although *irp_ang_* genes include most of the functions for piscibactin synthesis and utilization (Supplementary Table S1), the cluster lacks an *entD* homolog, which encodes a 4′-phosphopantetheinyl transferase, that is required to activate the peptide synthesis domains of non-ribosomal peptide synthetases (NRPS) (Crosa and Walsh, 2002). However, the *vab* gene cluster (**Figure 1A**), encoding the vanchrobactin system, does include a 4′-phosphopantetheinyl transferase gene (*vabD*) (Balado et al., 2008) that could complement *in trans* this function (see below).

In addition, there are three *orfs* (loci: VAR_RS19255, VAR_RS0101310, and VAR_RS19260), adjacent to *irp_ang_*, which encode a probable transposon belonging to the Tn7 superfamily (**Figure 1B**). The nucleotide sequence (accession No. AEZB01000007.1; region 10925–14484) including these three *orfs* shows a 86% nucleotide identity to the Tn7 transposon genes of the *V. parahaemolyticus* pathogenicity island (Vp-PAI) that encodes a type three secretion system (T3SSα) (Okada et al., 2009). Tn7 transposons are widespread in bacteria and are involved in the formation of some genomic islands that have the common feature of not being inserted near of tRNAs genes (Sugiyama et al., 2008). These findings suggest that *irp_ang_* could be part of a novel genomic island. Interestingly, a BLASTN search revealed that four of highly virulent *V. anguillarum* strains (two belonging to serotype O1 and two from serotype O2) (Rønneseth et al., 2017) contain the *irp_ang_* genes inserted in the same chromosomal location (Supplementary Figure S1). However, in two of these strains the *irp_ang_* genes are not associated with transposon genes (Supplementary Figure S1). The genome sequences of many *V. anguillarum* strains were recently reported (Busschaert et al., 2015; Castillo et al., 2017; Holm et al., 2018). Thus, we performed an *in silico* search within the 43 genomes available in GenBank and we could find the presence of *irp_ang_* gene cluster in several of these strains, some of which are highly virulent (**Table 3**). This fact suggests that the piscibactin cluster could have a relevant role in virulence.

**Table 3:** Distribution of vanchrobactin, piscibactin and anguibactin gene clusters among the *V. anguillarum* genome projects available in GenBank.

Strain	Serotype	Vanchrobactin	Piscibactin	Anguibactin	Assembly No.
775	O1	(+)	-	+	GCA_000217675.1
M3	O1	(+)	-	+	GCA_000462975.1
NB10	O1	(+)	-	+	GCA_000786425.1
90-11-286	O1	+	+	-	GCA_001660505.1
MVAV6203	O1	(+)	-	+	GCA_002163795.1
87-9-116	O1	(+)	-	+	GCA_002211505.1
JLL237	O1	+	+	-	GCA_002211985.1
S3 4/9	O1	+	+	-	GCA_002212005.1
VIB43	O1	+	+	-	GCA_002287545.1
ATCC-68554	O1	(+)	-	+	GCA_002291265.1
MVM425	O1	(+)	-	+	GCA_003031205.1
96F	O1	+	-	-	GCA_000257165.1
A023	O1	+	-	-	GCA_001989675.1
87-9-117	O1	(+)	-	+	GCA_001989715.1
91-8-178	O1	(+)	-	+	GCA_001989735.1
178/90	O1	(+)	-	+	GCA_001989755.1
261/91	O1	(+)	-	+	GCA_001989775.1
Ba35	O1	(+)	-	+	GCA_001989795.1
T265	O1	(+)	-	+	GCA_001989815.1
51/82/2	O1	(+)	-	-	GCA_001989855.1
LMG12010	O1	(+)	-	+	GCA_001989875.1
S2 2/9	O1	+	+	-	GCA_001989895.1
9014/8	O1	(+)	-	+	GCA_001989915.1
87-9-116	O1	(+)	-	-	GCA_001989935.1
90-11-287	O1	(+)	-	+	GCA_001990025.1
91-7154	O1	(+)	-	+	GCA_001990045.1
601/90	O1	(+)	-	+	GCA_001990065.1
6018/1	O1	(+)	-	+	GCA_001990085.1
VA1	O1	(+)	-	+	GCA_001990105.1
VIB93	O1	(+)	-	+	GCA_001990125.1
VIB18	O1	(+)	-	+	GCA_001998845.1
VIB12	O2	+	+	-	GCA_002310335.1
ATCC 14181	O2	+	-	-	GCA_001718015.1
DSM 21597	O2	+	+	-	GCA_001989995.1
M93	O2a	+	-	-	GCA_002901125.1
HI610	O2a	+	+	-	GCA_001989835.1
RV22	O2a	+	+	-	GCA_000257185.1
HI618	O2b	+	-	-	GCA_002078035.1
4299	O2b	+	-	-	GCA_001989655.1
CNEVA NB11008	O3	+	+	-	GCA_002212025.1
PF430-3	O3	+	-	-	GCA_001989695.1
PF7	O3	+	-	-	GCA_001997225.1
PF4	O3	+	-	-	GCA_002813835.1


***+** denotes the presence of a complete siderophore gene cluster. **(+)** denotes the insertion of an IS element into an essential biosynthetic gene. Gray shadows indicate the active siderophore systems in each strain.*

To test if *irp_ang_* genes are actually expressed, a series of reverse-transcriptase PCR reactions were conducted. The results showed that this gene cluster is transcribed in a polycistronic mRNA comprising *araC1*, *araC2*, *frpA*, *irp1*-*5*, *irp8* and *irp9* genes (**Figures 1B,C**). Therefore, the 10 genes putatively encoding piscibactin biosynthetic, regulatory, and uptake functions could be co-transcribed from the promoter upstream of *araC1*. A similar result was found for the piscibactin gene cluster harbored by *P. damselae* subsp. *piscicida* plasmid pPHDP70, which is transcribed in a single polycistronic mRNA from the promoter upstream of *dahP* (Osorio et al., 2006). Interestingly, the *irp_ang_* cluster does not include a *dahP* homolog and many other differences were found at the nucleotide level in intergenic regions. Thereby, even though *irp_ang_* genes can be transcribed from a promoter upstream of *araC1*, the existence of additional promoters, particularly upstream of *frpA*, cannot be ruled out (see below).

### *V. anguillarum* RV22 Produces Vanchrobactin and Piscibactin Simultaneously

**FIGURE 2:**
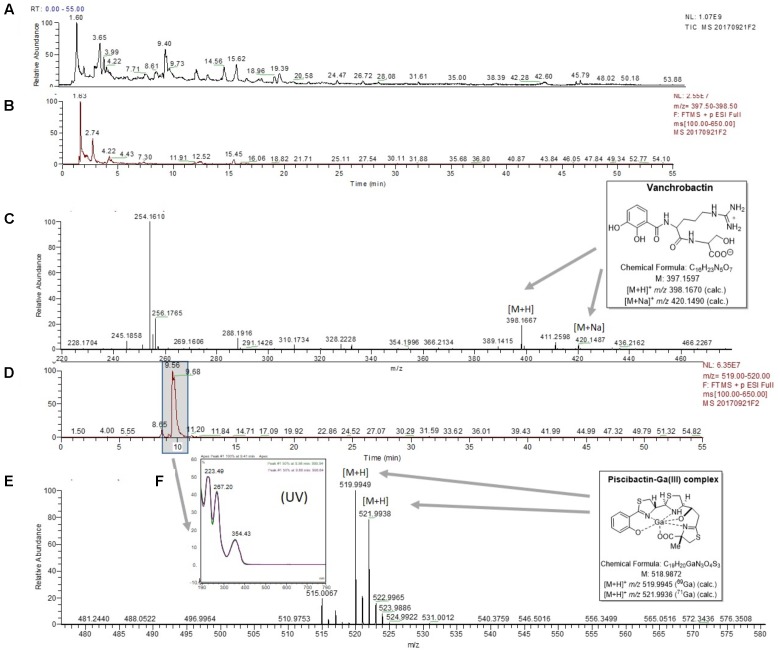
LC-MS experiments for the detection of vanchrobactin and piscibactin-Ga(III) complex in the RV22wt strain. **(A)** Total ion chromatogram (TIC) of the fraction RV22WTF3 eluted with H_2_O-CH_3_CN (1:1) from the Oasis HLB cartridge. **(B)** Extracted mass chromatogram (*m/z* 397.50 to 398.50) showing the peak with retention time of 2.74 min. **(C)** (+)-HRESIMS of the peak at *t_R_* of 2.74 min identified as vanchrobactin: *m/z* 398.1667 ([M+H]^+^; calc. for C_16_H_24_N_5_O_7_, 398.1670) and *m/z* 420.1487 ([M+Na]^+^; calc. for C_16_H_23_N_5_O_7_Na, 420.1490). **(D)** Extracted mass chromatogram (*m/z* 519.00 to 520.00) showing the peak with retention time of 9.56 min. **(E)** (+)-HRESIMS of the peak at *t_R_* of 9.56 min identified as piscibactin-Ga(III) complex: *m/z* 519.9946/521.9933 ([M+H]^+^, calc. for C_19_H_21_N_3_O_4_S_3_Ga, 519.9950/521.9941). **(F)** UV spectrum of the peak at *t_R_* of 9.56 min.

To demonstrate that RV22 synthesizes piscibactin in addition to vanchrobactin (Soengas et al., 2006, 2007), we used the previously described methodology, based on the use of HLB cartridges and liquid chromatography-mass spectrometry (LC-MS), that we developed for isolation of piscibactin (Souto et al., 2012). Vanchrobactin was first detected with a retention time (*t_R_*) of 2.74 min (**Figures 2A,B**), which showed the [M+H]^+^ and [M+Na]^+^ ions at *m/z* 398.1667 and 420.1487, respectively (**Figure 2C**). Furthermore, from the cell-free supernatants of RV22 cultures under iron restriction and after addition of GaBr_3_, in order to stabilize the siderophore as its corresponding complex, it was detected the characteristic piscibactin-Ga(III) complex with (*t_R_*) of 9.56 min (**Figure 2D**). The chemical identification was done on the basis of its *t_R_* value, UV spectral data and HRESIMS analysis. Thus, piscibactin-Ga(III) complexes obtained from *V. anguillarum* RV22 and from *P. damselae* subsp. *piscicida* (Souto et al., 2012) showed similar retention times and absorbance maxima (223, 267, and 354 nm) in its UV spectrum (**Figure 2F**) in HPLC analysis. Moreover, HRESIMS analyses showed the [M+H]^+^ ions at *m/z* 519.9949/521.9938 (calculated for C_19_H_21_N_3_O_4_S_3_Ga, 519.9950/521.9941), with the distinctive isotopic ratio of gallium (Mr = 69 and 71, ratio 3:2) (**Figure 2E**), indicating the same molecular mass to that reported for piscibactin-Ga (III) complex isolated from *P. damselae* subsp. *piscicida* (Souto et al., 2012).

**FIGURE 3:**
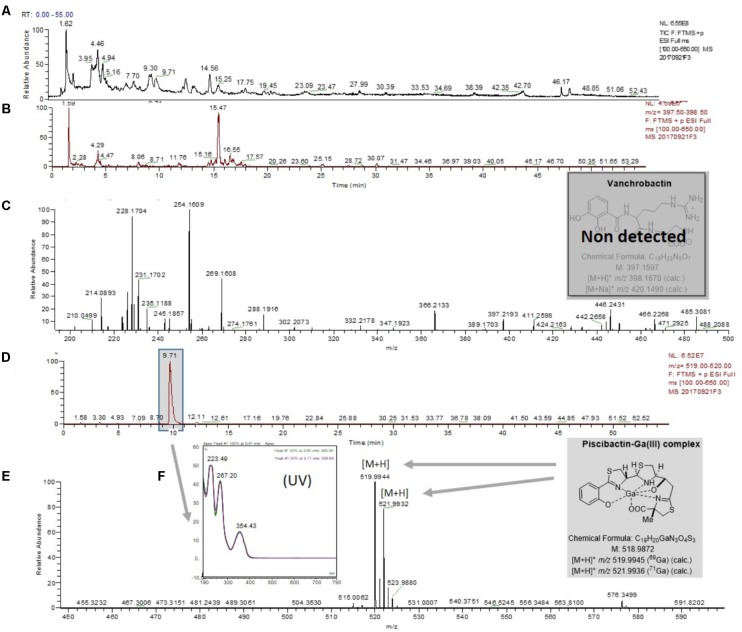
LC-MS experiments for the detection of piscibactin-Ga(III) complex in the RV22Δ*vabF* mutant strain. **(A)** Total ion chromatogram (TIC) of the fraction RV22Δ*vabF*F3 eluted with H_2_O-CH_3_CN (1:1) from the Oasis HLB cartridge. **(B)** Extracted mass chromatogram (*m/z* 397.50 to 398.50). **(C)** (+)-HRESIMS corresponding to retention time window from *t_R_* of 0.19–3.00 min which shows the lack of vanchrobactin. **(D)** Extracted mass chromatogram (*m/z* 519.00 to 520.00) showing the peak with retention time of 9.71 min. **(E)** (+)-HRESIMS of the peak at *t_R_* of 9.71 min identified as piscibactin-Ga(III) complex: *m/z* 519.9944/521.9922 ([M+H]^+^, calc. for C_19_H_21_N_3_O_4_S_3_Ga, 519.9950/521.9941). **(F)** UV spectrum of the peak at *t_R_* of 9.71 min.

As expected, chemical analysis of cell-free supernatants of the *V. anguillarum* RV22Δ*vabF* mutant (unable to produce vanchrobactin), following the same methodology, showed the absence of vanchrobactin (**Figures 3A–C**) and the presence of a peak with a *t_R_* of 9.71 min (**Figure 3D**), displaying a *m/z* value of 519.9945/521.9933 (**Figure 3E**) and a UV spectrum (**Figure 3F**) which correspond to piscibactin-Ga(III) complex. The same methodology was used to test piscibactin synthesis by *V. anguillarum* RV22Δ*vabD* mutant (Balado et al., 2008), which lacks an active 4′-phosphopantetheinyl transferase. In this mutant, neither vanchrobactin or piscibactin were detected (Supplementary Figure S2), confirming the *vabD* gene is essential for the synthesis of both siderophores.

**FIGURE 4:**
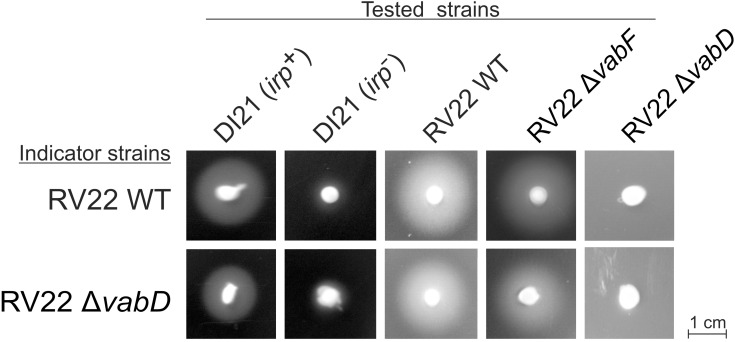
Cross feeding assay to determine the production and use of piscibactin by *V. anguillarum* RV22 wild type and RV22Δ*vabD* mutant. Indicator strains are those inoculated within the CM9 plates containing 120 μM 2,2′-dipyridyl. Tested strains are those tested by placing a loopful of bacterial biomass on the agar surface. A growth halo indicates that indicator strains can use the siderophores produced by tested strains to overcome the iron limitation conditions. *Photobacterium damselae* subsp. *piscicida* DI21 wild type strain (irp^+^, piscibactin producer) and DI21 cured from plasmid pPHDP70 (irp^-^, unable to produce piscibactin) were used as controls. RV22Δ*vabF* does not produce vanchrobactin; RV22Δ*vabD* does not produce vanchrobactin nor piscibactin.

In addition, cross-feeding assays were used to ascertain piscibactin production and utilization by *V. anguillarum* RV22 (**Figure 4**). RV22 wild type and RV22Δ*vabD* mutant could be cross-fed by a piscibactin-producing strain (*P. damselae* subsp. *piscicida* DI21). The *V. anguillarum* mutant RV22Δ*vabF* (impaired for vanchrobactin production) still could cross-fed both indicator strains, indicating the production of a second siderophore. However, no growth halo was obtained when using RV22Δ*vabD* mutant as tested strain (**Figure 4**). All these results prove that *V. anguillarum* RV22 is able to produce and use piscibactin, which denotes that *irp_ang_* genes are functional. Moreover, the results clearly suggest that *irp_ang_* gene cluster needs a functional *vabD* gene (encoding the 4′-phosphopantetheinyl transferase) to complete the biosynthesis of piscibactin.

### Piscibactin Synthesis Contributes to the Growth Ability Under Iron Restriction Depending of Environmental Conditions

**FIGURE 5:**
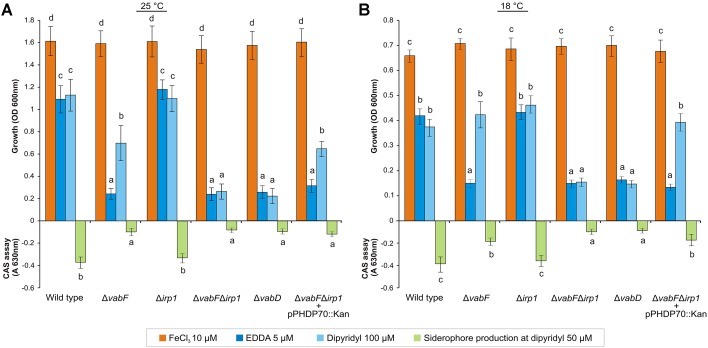
Growth of *V. anguillarum* strains cultivated for 24 h under iron excess or iron starvation in CM9 medium and incubated at 25°C **(A)** or 18°C **(B)**. Siderophore production (indicated as A_630_ values), determined by the CAS test performed on culture supernatants, is also displayed for all strains. In each panel, bars labeled with the same letter (a, b, c, d) are not significantly different from each other (*t*-test). Error bars denote standard deviations.

To test whether *irp1* is required for piscibactin production and to study the influence of piscibactin on *V. anguillarum* cell fitness, in-frame deletion mutants of *irp1* were obtained in RV22wt and RV22Δ*vabF* (deficient in vanchrobactin synthesis) backgrounds. The growth ability of resultant RV22Δ*irp1* and RV22Δ*vabF*Δ*irp1* mutant strains was measured at 25°C under iron excess and under iron-deprivation conditions using the strong iron-chelating agent ethylenediamine-di-(*o*-hydroxyphenyl-acetic acid) (EDDA), or a weaker one, 2,2′-dipyridyl. RV22 wild type strain (that produces both vanchrobactin and piscibactin) was used as positive growth control showing a minimal inhibitory concentration (MIC) of EDDA and 2,2′-dipyridyl of 10 μM and 150 μM, respectively. Conversely, RV22Δ*vabD* strain (Balado et al., 2008), impaired to produce any of these siderophores, showed MICs of 5 μM for EDDA and 100 μM for 2,2′-dipyridyl. When the mutants were cultured under iron-excess conditions (CM9 plus 10 μM ferric chloride) no significant differences in growth levels were observed with respect to the wild type strain (**Figure 5A**). However, significant differences were observed between RV22wt and some of the siderophore mutants when cultivated under iron deficiency. When EDDA (CM9 plus 5 μM EDDA) was used as chelating agent, the RV22Δ*irp1* mutant (producing only vanchrobactin) and the parental strain showed identical growth levels. However, the mutant producing only piscibactin (RV22Δ*vabF*) was unable to grow (**Figure 5A**). These results corroborate those previously published (Balado et al., 2006) and confirm that inactivation of vanchrobactin synthesis strongly reduce the growth ability of *V. anguillarum* under *in vitro* iron starvation. Interestingly, when growth was measured in CM9 containing 100 μM of the chelator 2,2′-dipyridyl the mutant RV22Δ*vabF* reached around 50% of the growth levels showed by the parental strain (**Figure 5A**). Siderophore-like activity present in cell free supernatants, measured by the CAS assay after growing each *V. anguillarum* strain at 50 μM 2,2′-dipyridyl, correlated with the growth levels observed.

The same growth experiments were conducted also at 18°C since it is a temperature closer to the conditions encountered *in vivo* during infection. At this temperature of incubation, and in presence of 100 μM 2,2′-dipyridyl, RV22Δ*vabF* (producing only piscibactin) behaved similarly to the wild type strain (**Figure 5B**), which suggests that piscibactin production by *V. anguillarum* could be favored at low temperatures (see below). Reactivation of piscibactin synthesis in RV22Δ*vabF*Δ*irp1* double mutant by introduction of pPHDP70 plasmid (Osorio et al., 2015), resulting in strain RV22Δ*vabF*Δ*irp1*(pPHDP70), showed a phenotype almost identical to RV22Δ*vabF* strain in both temperatures assayed (**Figures 5A,B**).

Overall, these results suggest that (i) *V. anguillarum* may use vanchrobactin as primary siderophore and that (ii) piscibactin efficacy to provide iron to support growth depends on the environmental conditions, in particular on the iron source and the temperature. Additionally, the results also suggest that the role of vanchrobactin in the biology of *V. anguillarum* could be previously overestimated (Balado et al., 2006) probably owing to the fact that vanchrobactin defective mutants have been studied only under stringent conditions of iron limitation by the use of EDDA as iron chelator.

### Piscibactin Contributes to a Greater Extent Than Vanchrobactin to Virulence for Turbot

**FIGURE 6:**
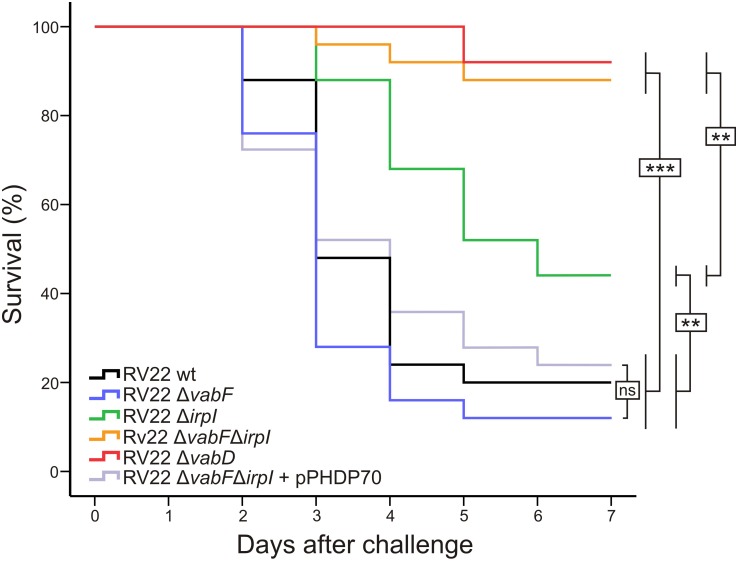
Percentage survival after 7 days of turbot fingerlings challenged with 2–4 × 10^4^ CFU/fish of *V. anguillarum* RV22 wild type strain or with the siderophore mutants analyzed. Asterisks denote statistically significant differences between strains: ^∗^*P* < 0.05; ^∗∗^*P* < 0.01; ^∗∗∗^*P* < 0.001; ns, no statistically significant differences.

In order to study the contribution of each siderophore (vanchrobactin and piscibactin) to *V. anguillarum* pathogenesis, the virulence of the RV22 wild type strain, as well as of the mutants deficient in one of the two siderophores or in both of them, was evaluated by experimental infections in turbot. The fish were kept at 18–20°C and were inoculated intraperitoneally with 2–4 × 10^4^ CFU of the wild type or with one of the single or double siderophore mutants. The wild type strain RV22 killed 80% of the fish after 5 days of challenge (**Figure 6**). When the challenge was done with the mutant that produces only vanchrobactin (RV22Δ*irp1*) the mortality reached 56% after 6 days of infection. However, when the fish were inoculated with the mutant that produces only piscibactin (RV22Δ*vabF*) the mortality reached 88% after 5 days, at the same level of the wild type strain. The double mutant RV22Δ*vabF*Δ*irp1* and the RV22Δ*vabD* (both unable to produce any of the two siderophores) give mortalities of 12% and 8%, respectively, after 7 days. Interestingly, when piscibactin production was reactivated by the introduction of pPHDP70 plasmid (that contains the piscibactin synthesis gene cluster) (Osorio et al., 2015) into RV22Δ*vabF*Δ*irp1* double mutant, this complemented strain caused a mortality of 78% after 6 days (**Figure 6**). These results clearly demonstrate that the ability to synthesize a siderophore has a great impact in the virulence of *V. anguillarum*. However, each siderophore system contributes in different grade to virulence being piscibactin production, more than vanchrobactin, the main virulence factor in the tested conditions.

### Transcriptional Analysis of the *irp_ang_* Gene Cluster: The Expression of Piscibactin Genes Are Favored at Low Temperatures

**FIGURE 7:**
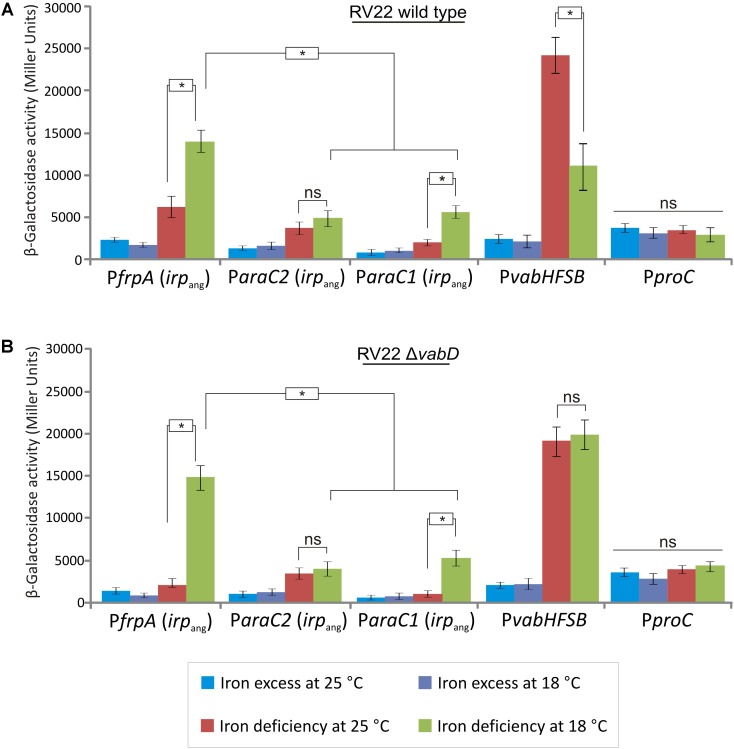
Transcriptional activity (β-galactosidase units) of the *lacZ* fusions to the P1, P2 and *vabH* promoters in *V. anguillarum* RV22wt **(A)** or RV22*ΔvabD* mutant impaired to produce siderophores **(B)** grown at 25°C or 18°C in CM9 medium under different iron availability conditions: iron excess and CM9 containing 50 μM 2,2′-dipyridyl. *lacZ* fusion with the constitutive promoter of *proC* (P*proC*) was used as control. Asterisks indicate statistically significant differences between bars (*P* < 0.05); ns, no statistically significant differences. Error bars denote standard deviations.

The synthesis of siderophores are tightly regulated at transcriptional level which ensures that siderophore production occurs only when it is necessary (Cornelis et al., 2011). In order to analyze the expression levels of the *irp_ang_* putative promoters, DNA fragments of ca. 700 nucleotides upstream *frpA_ang_*, *araC1_ang_* and *araC2_ang_* genes, that were named P*frpA* P*araC1* and P*araC2*, respectively (**Figure 1B**), were cloned into the plasmid pHRP309 upstream of a promoterless *lacZ* gene. *frpA* encodes the presumptive ferri-piscibactin outer membrane receptor while *araC1* and *araC2* encode two putative AraC-type transcriptional regulators (Supplementary Table S1). Resulting plasmids were mobilized into *V. anguillarum* RV22wt and the transcription levels were measured by determining β-galactosidase activities under different iron availability conditions at 25°C (**Figure 7A**). The expression levels were compared with the promoter of the vanchrobactin system located upstream of *vabH* (Balado et al., 2008). Interestingly, the use of the P*frpA*, P*araC1*, and P*araC2* presumptive promoters produced significant β-galactosidase activity (**Figure 7**) which demonstrates that the three sequences serve as transcriptional starts. In addition, under the iron-restricted conditions tested, the expression from promoter P*frpA* was three-times higher than from P*araC1* or P*araC2* (**Figure 7A**). Thus, although the transcription of piscibactin genes could start from any of the three promoters, it seems to occur to a greater extent from the promoter immediately upstream of *frpA* (P*frpA*), which encodes the probable piscibactin receptor.

Usually, expression of siderophore systems promoters are down-regulated under iron availability (Cornelis et al., 2011). As expected, the addition of an iron chelator to the medium produced a significant increase of P*frpA*, P*araC1*, and P*araC2* activities (**Figure 7A**). However, the maximal expression levels achieved by piscibactin promoters under high iron deprivation (by addition of 2,2′-dipyridyl 100 μM) at 25°C were fourfold lower with respect to the vanchrobactin promoter (**Figure 7A**). These findings strongly contrast with the high relevance of piscibactin in virulence, as demonstrated above (**Figure 6**). This low expression at 25°C could be due to: (1) *irp*_ang_ genes could remain down-regulated because enough iron is entering the cell through the vanchrobactin system; or (2) piscibactin promoters need additional environmental signals, not only low iron levels, to achieve their maximal activity. These two hypothesis need to be further studied.

Since, as shown above, temperature could have a relevant role in piscibactin production, we wanted to ascertain if the expression levels of piscibactin genes could be favored at low temperatures. For this purpose, transcriptional levels of the piscibactin promoters were measured in RV22wt strain incubated at 18°C and compared to those observed at 25°C. While P*araC2* showed almost the same activity at both temperatures, we found an increment of ca. twofold in the P*frpA* and P*araC1* activity at 18°C with respect to that measured at 25°C (**Figure 7A**). In contrast, the activity of the vanchrobactin promoter decreased twofold. When constitutive *proC*-promoter was tested, no significant differences in *lacZ* activity were observed between conditions assayed either temperature or iron availability (**Figures 7A,B**). These findings show that expression of piscibactin genes are favored when *V. anguillarum* grows at low temperatures. In addition, the increase of piscibactin promoters expression, together with the decrease of vanchrobactin promoters at low temperatures, strongly suggests that *V. anguillarum* is able to balance the synthesis of each siderophore responding to environmental signals like iron levels and temperature (**Figure 7**). To ascertain if the decrease in vancrobactin promoter activity at 18°C was due to the temperature or if it was caused by the intake of iron mediated by piscibactin, the expression levels of the three promoters were measured in the RV22*ΔvabD* strain, which is unable to produce any siderophore. While P*frpA* and P*araC1* piscibactin promoters showed a strong temperature-dependent expression pattern again, the vanchrobactin promoter (P*vabH*) showed almost identical β-galactosidase activity at 25 or 18°C (**Figure 7B**). These results suggest that the lower activity of vanchrobactin promoter at 18°C in the RV22wt strain may be due to the increased intake of iron through the piscibactin system more than to a temperature-dependent regulation. Overall, these results strongly suggest that the expression of *irp*_ang_ genes in *V. anguillarum* are favored at low growth temperatures.

## Discussion

In the present work, we could identify in *V. anguillarum* a siderophore gene cluster, named *irp_ang_*, that is contained within a pathogenicity island and that shows a strong homology to its counterpart described in the pPHDP70 plasmid from *P. damselae* subsp. *piscicida* (Osorio et al., 2015). This PAI is located in the second chromosome of some highly pathogenic strains of *V. anguillarum*, including strain RV22, from which vanchrobactin genes were previously identified (Balado et al., 2006, 2008). The *irp_ang_* cluster shows the same genetic organization than the *irp* genes of *P. damselae* subsp. *piscicida* and both confer the ability to produce the siderophore piscibactin. The PAI island harboring the piscibactin genes in *P. damselae* subsp. *piscicida* encodes all functions required to produce and use piscibactin (Osorio et al., 2015). However, piscibactin synthesis in *V. anguillarum* depends on the presence in the recipient genome of a functional 4′-phosphopantetheinyl transferase gene (*entD*). A similar case was reported for *Aeromonas salmonicida*, where acinetobactin and amonabactin biosynthetic pathways share the same set of genes encoding the biosynthesis of 2,3-DHBA, a common moiety of both siderophores, and also share an EntD homolog (Balado et al., 2015).

Chemical analysis demonstrated that piscibactin is indeed being synthesized by the vanchrobactin-producing *V. anguillarum* strains. Although the production of more than one siderophore by a bacterial cell could be deleterious (Cordero et al., 2012), in some cases the ability to produce two siderophores can enhance niche flexibility (Sandy et al., 2010; Dumas et al., 2013) and pathogenesis (Garcia et al., 2011). Some pathogens carry multiple iron acquisition systems that seem redundant in laboratory culture conditions. For instance, the fish pathogen *Aeromonas salmonicida* subsp. *salmonicida* produces acinetobactin and amonabactin, two catechol siderophores and, although there are strains that produce both siderophores simultaneously, most strains have the amonabactin cluster inactivated (Balado et al., 2015). By contrast, the co-expression of siderophores with different chemical properties can play specialized roles at the host-pathogen interface (Fetherston et al., 2010; Koh and Henderson, 2015). Piscibactin, along with yersiniabactin and pyochelin, constitute a group of siderophores that share similar structural characteristics (Crosa and Walsh, 2002; Souto et al., 2012). It has been shown that the co-expression of yersiniabactin with other catecholate siderophores enhances the virulence properties of uropathogenic *Escherichia coli* (Garcia et al., 2011). Yersiniabactin and pyochelin seem to play several roles, other than iron uptake, during infection since they can efficiently bind other metals besides iron. For instance, yersiniabactin participates in zinc (Zn^+2^) acquisition and also confers protection against copper toxicity, which promotes bacterial colonization, dissemination and resistance against phagocytosis (Fetherston et al., 2010; Chaturvedi et al., 2012; Bobrov et al., 2014; Koh and Henderson, 2015; Holden et al., 2016). Pyochelin has been also associated with some traits such as oxidative or inflammatory activities in *Pseudomonas aeruginosa* infections (Britigan et al., 1997), decomposition of organotin compounds (like TPT, triphenyltin chloride or DPT, diphenyltin dichloride) (Sun et al., 2006) or plants defense against fungal plagues (Audenaert et al., 2002). Some of these or other related roles could also be attributed to piscibactin in *V. anguillarum*.

Interestingly, the inactivation of piscibactin synthesis in *V. anguillarum* RV22 produced just a small decrease in the growth ability under iron restriction. This suggests that these mutants would use vanchrobactin to internalize iron. Thus, vanchrobactin would serve *in vitro* as an efficient iron carrier (Iglesias et al., 2011) for *V. anguillarum* while the piscibactin efficacy to supply iron to the cell would depend on which is the iron source available (**Figure 5**). However, this observation highly contrasts with our results of the virulence assays where piscibactin production alone was sufficient to obtain the maximal virulence. The inactivation of piscibactin system resulted in a severe loss of the virulence degree even if vanchrobactin system was functional (**Figure 6**). Hence, although the ability to synthesize one siderophore, either vanchrobactin or piscibactin, has a great impact in *V. anguillarum* virulence, the ability to produce piscibactin seems to be sufficient to confer maximal virulence to *V. anguillarum*, at least in the tested conditions.

A total of 43 *V. anguillarum* genomes are currently available in the genomic databases and the virulence properties of 32 of these strains were recently reported (Busschaert et al., 2015; Rønneseth et al., 2017). An *in silico* analysis showed that many of these virulent *V. anguillarum* strains harbor both, vanchrobactin and piscibactin, gene clusters (**Table 3**). Thus, piscibactin genes are widespread in *V. anguillarum* strains and they are associated with a highly virulent phenotype for several fish species, such as turbot (*Psetta maxima*), cod (*Gadus morhua*), halibut (*Hippoglossus hippoglossus*), rainbow trout (*Oncorhynchus mykiss*) or sea bass (*Dicentrarchus labrax*) (Busschaert et al., 2015; Rønneseth et al., 2017). These results support the previously suggested hypothesis that vanchrobactin is the ancient siderophore of *V. anguillarum* (Lemos et al., 2010). However, according to our results and to previously published works (Sandy et al., 2010), vanchrobactin production could be more related to the persistence of *V. anguillarum* in the marine environment than to pathogenesis. Hence, piscibactin would be the most important siderophore during the vibriosis outbreaks in fish. Interestingly, it was recently found that piscibactin is one of the most extended siderophore systems in *Vibrionaceae* family. Phylogenetic analysis of three siderophore systems (piscibactin, vibrioferrin, and aerobactin) suggested that their current distribution could be explained by an old insertion that was followed by the action of diverse evolutionary forces (Thode et al., 2018). An interesting observation is that the piscibactin gene cluster is never present in anguibactin-producing strains (**Table 3**). It has been previously reported that the presence of pJM1-like plasmids encoding the anguibactin system inactivates the vanchrobactin cluster through a transposition event (Naka et al., 2008). A similar genetic incompatibility could explain the mutual exclusion between piscibactin and anguibactin gene clusters.

Many of the genes encoding virulence factors, including iron uptake systems, are preferentially expressed within the host in response to particular environmental signals (Lee and Camilli, 2000). The analysis of promoters activity showed that *irp*_ang_ genes in *V. anguillarum* are not only strongly iron-regulated, but also, that piscibactin production has a requirement for low temperatures. These findings suggest that *V. anguillarum* is able to modulate the synthesis of vanchrobactin and piscibactin according to the surrounding temperature, which adjust the energy costs of siderophore production to the environmental circumstances. Some bacterial species that are able to produce more than one siderophore can modulate the expression as a response to environmental factors, e.g., pH, carbon source, amino sugars or temperature (Valdebenito et al., 2006; Craig et al., 2012; Cornelis and Dingemans, 2013; Dumas et al., 2013; Tanabe et al., 2014). The temperature-dependent regulation is characteristic of pathogenic bacteria affecting ectothermic hosts such as fish, where the infection processes generally occur at temperatures lower than those required for bacterial optimal growth (Guijarro et al., 2015). However, these regulation mechanisms are yet poorly understood and few examples were reported of siderophore synthesis regulated by temperature. One of these is ruckerbactin, a catecholate siderophore produced by *Yersinia ruckeri* (Fernández et al., 2004); other one is the hydroxamate bisucaberin produced by *Aliivibrio salmonicida*, the causative agent of cold-water vibriosis (Colquhoun and Sørum, 2001; Winkelmann et al., 2002). In both bacteria, the maximal siderophore production take place around the temperatures at which the diseases outbreaks occur. Our results also suggest that piscibactin synthesis in *V. anguillarum* is higher at temperatures (16–19°C) at which the vibriosis outbreaks usually occur in turbot (Toranzo et al., 2005, 2017).

## Conclusion

Pathogenic *V. anguillarum* strains lacking the anguibactin system produce two chromosomally encoded siderophores, vanchrobactin and piscibactin. While vanchrobactin synthesis seems to be regulated only by the iron availability, piscibactin is preferentially produced at low temperatures at which fish vibriosis outbreaks usually occur in natural conditions. Piscibactin seems to contribute to a greater extent than vanchrobactin to the virulence for turbot. Further studies are currently under way to decipher the regulation pathways that control piscibactin synthesis in *V. anguillarum*.

## Author Contributions

MB, JR, CJ, and MLL contributed to the conception and design of the study. MB, MAL, JF-M, and DM-M performed the lab experiments. MB, CJ, and MLL analyzed the data. MB wrote the first draft of the manuscript. CJ, JR, and MLL corrected the draft and build the final version of the manuscript. All authors contributed to manuscript revision, read and approved the submitted version.

## Conflict of Interest Statement

The authors declare that the research was conducted in the absence of any commercial or financial relationships that could be construed as a potential conflict of interest.

## Footnotes

1 http://www.prodoric.de
