# Supplementary material for: The Siderophore Piscibactin Is a Relevant Virulence Factor for Vibrio anguillarum Favored at Low Temperatures
Source: Front Microbiol. 2018 Aug 2;9:1766. doi: 10.3389/fmicb.2018.01766 (PMC6083037; doi:10.3389/fmicb.2018.01766)
Supplement: Supplementary file 1 [file Data_Sheet_1.PDF]

Supplementary Information

The siderophore piscibactin is a relevant virulence factor for *Vibrio anguillarum* favoured at low temperatures

Miguel Balado[a], Marta A. Lages[a], Juan C. Fuentes-Monteverde[b], Diana Martínez-Matamoros[b], Jaime Rodríguez[b], Carlos Jiménez[b], Manuel L. Lemos[a*]

[a] Department of Microbiology and Parasitology, Institute of Aquaculture, Universidade de Santiago de Compostela, Santiago de Compostela 15782, Spain.
[b] Department of Chemistry, Faculty of Sciences and Center for Advanced Scientific Research (CICA), Universidade da Coruña, A Coruña 15071, Spain.

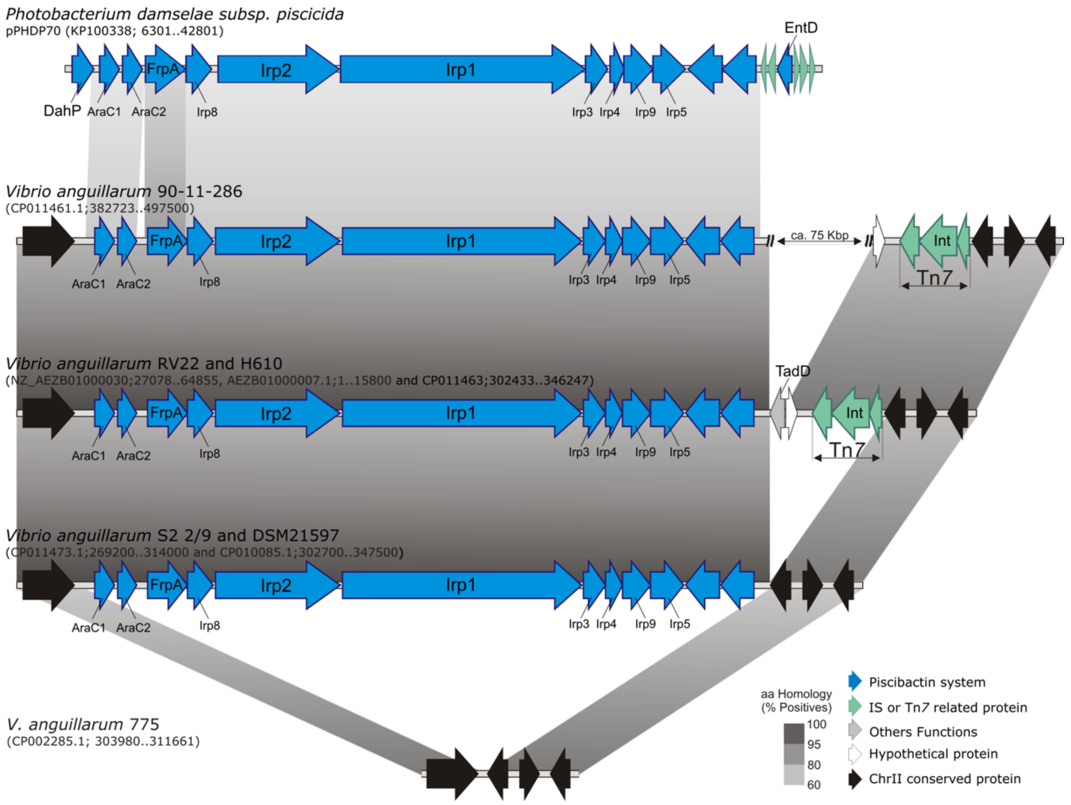

Fig. S1. Homology of *P. damselae* subsp *piscicida* gene cluster encoding piscibactin synthesis and transport with the irp$_{ang}$ gene cluster present in several strains of *V. anguillarum*.

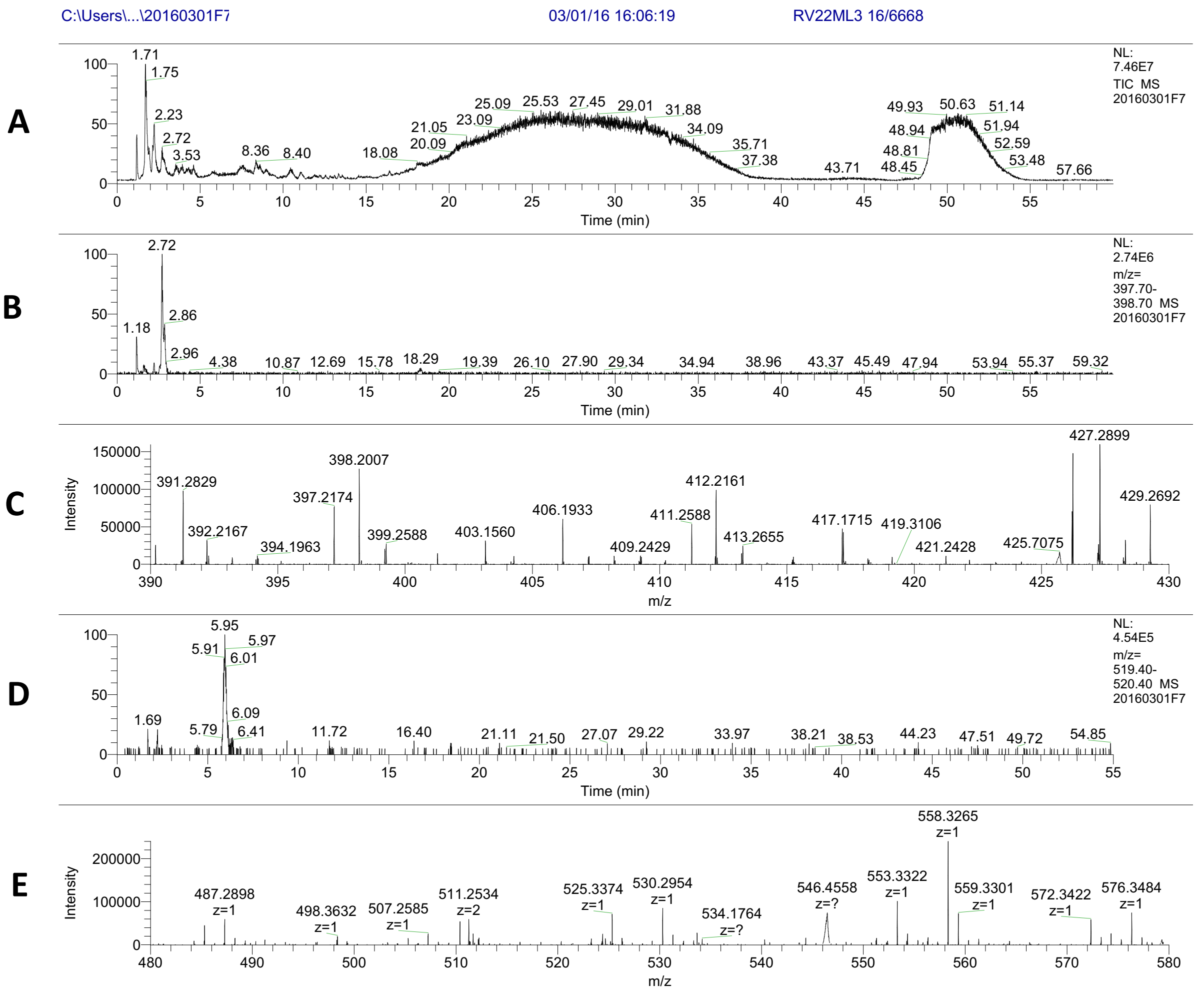

Fig S2. LC-MS experiments for the detection of vanchrobactin and piscibactin-Ga(III) complex in the RV22Δ*vabD* mutant strain unable to produce vanchrobactin or piscibactin. (**A**) Total ion chromatogram (TIC) of the fraction RV22 eluted with H_2O-CH_3CN (1:1) from the Oasis HLB cartridge; (**B**) Extracted mass chromatogram (*m/z* 397.70 - 398.70); (**C**) (+)-HRESIMS corresponding to retention time (t_R) window from 2.0 to 5.0 min which shows the absence of vanchrobactin; (**D**) Extracted mass chromatogram (*m/z* from 519.40 - 520.40) (**E**) (+)-HRESIMS corresponding to retention time window from of 5.0 to 15.0 min which shows the absence of piscibactin-Ga(III) complex. Note: Both retention time windows were chosen according to the retention time expected for each compound.

Table S1. Function of *irp_{ang}* genes and their homology to piscibactin genes of *Photobacterium damselae* subsp. *piscicida*.

Protein ID, No aa	Name, description	Irp cluster homologues of *PDP* [a] (Acc. No, Homology identities-positives)
WP_019281874.1, 326	AraC1, putative AraC transcriptional regulator	(AKQ52527.1, 59-76)
WP_019281875.1, 292	AraC2, Putative AraC transcriptional regulator	(AKQ52528.1, 59-75)
WP_019281876.1, 647	FrpA, TonB-dependent receptor	(AKQ52529.1, 66-81)
WP_019281877.1, 422	Irp8, major facilitator superfamily protein	(AKQ52530.1, 59-73)
WP_019281878.1, 2064	Irp2, nonribosomal peptide synthetase	(AKQ52531.1, 57-70)
WP_019281879.1, 3967	Irp1, nonribosomal peptide synthetase	(AKQ52532.1, 52-66)
WP_019281880.1, 366	Irp3, putative reductase component	(AKQ52533.1, 61-74)
WP_019281881.1, 290	Irp4, thioesterase protein	(AKQ52534.1, 54-65)
WP_019281882.1, 464	Irp9, salicylate synthase	(AKQ52535.1, 61-72)
WP_019281883.1, 552	Irp5, 2,3-dihydroxybenzoate-AMP ligase	(AKQ52536.1, 63-78)
WP_019281884.1, 565	ABC transporter ATP-binding protein	(AKQ52537.1, 59-75)
WP_017046020.1[b], 551	ABC transporter transmembrane region	(AKQ52538.1, 58-75)

[a] *PDP*: *Photobacterium damselae* subsp. *piscicida*

[b] Id. of its identical protein group since this gene is not annotated in the genome of strain RV22.
